# Exploring the binding mechanisms of PDE5 with chromeno[2,3-*c*]pyrrol-9(2*H*)-one by theoretical approaches†

**DOI:** 10.1039/c8ra06405a

**Published:** 2018-08-29

**Authors:** Xianfeng Huang, Peng Xu, Yijing Cao, Li Liu, Guoqiang Song, Lei Xu

**Affiliations:** School of Pharmaceutical Engineering and Life Sciences, Changzhou University Changzhou Jiangsu 213164 PR China drugs@vip.sina.com +86-519-86334598 +86-519-86330600; Department of Orthopedics, Second Military Medical University Affiliated Changzheng Hospital Shanghai 200003 China; Institute of Bioinformatics and Medical Engineering, School of Electrical and Information Engineering, Jiangsu University of Technology Changzhou 213001 China leixu@jsut.edu.cn +86-519-86953223 +86-519-86953220

## Abstract

Cyclic nucleotide phosphodiesterase type 5 (PDE5), exclusively specific for the cyclic guanosine monophosphate (cGMP), is an important drug target for the treatment of erectile dysfunction and pulmonary arterial hypertension (PAH). Although many PDE5 inhibitors have been approved, such as sildenafil, vardenafil, tadalafil and so on, extensive studies have reported some side effects, such as vision disturbance and hearing loss as a result of the amino acid sequence and the secondary structural similarity of other PDEs to the catalytic domain of PDE5. In this study, multiple docking strategies, molecular dynamics (MD) simulations, free energy calculations and decomposition were employed to explore the structural determinants of PDE5 with a series of chromeno[2,3-*c*]pyrrol-9(2*H*)-one derivatives. First, reliable docking results were obtained using quantum mechanics/molecular mechanics (QM/MM) docking. Then, MD simulations and MM/GBSA free energy calculations were used to explore the dynamic binding process and characterize the binding modes of the inhibitors with different activities. The predicted binding free energies are in good agreement with the experimental data, and the MM/GBSA free energy decomposition analysis sheds light on the importance of hydrogen bonds with Gln817, π–π stacks against Phe820 and hydrophobic residues for the PDE5 binding of the studied inhibitors. The structural and energetic insights obtained here are useful for understanding the molecular mechanism of ligand binding and designing novel potent and selective PDE5 inhibitors with new scaffolds.

## Abstract 2

Cyclic nucleotide phosphodiesterase type 5 (PDE5) is exclusively specific for the cyclic guanosine monophosphate (cGMP), and PDE5 is an important drug target for the treatment of erectile dysfunction and pulmonary arterial hypertension (PAH).

## Graphical abstract

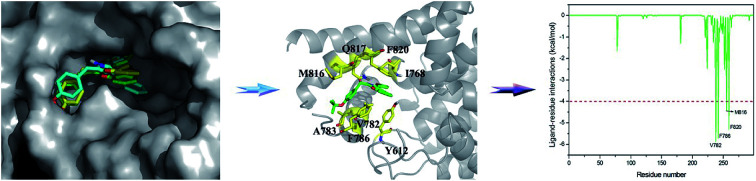

## Introduction

Cyclic nucleotide phosphodiesterases (PDEs) can hydrolyze cellular adenosine and guanosine 3′,5′-cyclic monophosphate (cAMP and cGMP), which are important secondary messengers mediating many physiological processes, including cardiac and smooth muscle contraction, inflammation, circadian regulation and so on.^1,2^ Owing to the important roles of cAMP and cGMP, PDE inhibitors have been applied in a wide range of human therapeutic areas, such as diabetes,^3^ Alzheimer's disease,^4^ asthma,^5^ and erectile dysfunction.^6^ To date, there are 11 human PDE families (PDE1-11), of which PDE5, a cGMP-specific enzyme, is the most successful target for the development of inhibitors to treat male erectile dysfunction (ED) and cardiovascular diseases.^7^ The solved crystal structures show that PDE5 is homodimer consisting of two regulatory GAF domains at the N-terminus, a phosphorylation site at the Ser92 position and a catalytic site at the C-terminal end (amino acid residues: 535–860).^8^ All inhibitors are located at the substrate binding pocket of the PDE5 catalytic domain, which are composed of 16 helices (H1–H16) and 16 loops (A–N). Currently, several PDE5 inhibitors have been approved by the FDA for the treatment of erectile dysfunction (sildenafil, vardenafil, tadalafil, avanafil, udenafil, and mirodenafil) and pulmonary arterial hypertension (PAH) (sildenafil and tadalafil).^9^ Moreover, extensive studies have revealed that PDE5 inhibitors are potent for the treatment of other diseases, such as lower urinary tract symptoms (LUTS),^10^ heart failure and coronary artery disease,^11^ neurological disorders^12^ and so on. However, preclinical and clinic trials have reported some side effects, such as vision disturbance and hearing loss, which stemmed from the cross-reactivity of these drugs against other PDE families or poor responses in some patients. Great attention of both academic and industrial researchers is focused on the development of the second generation PDE5 inhibitors with better selectivity against other enzymes within the family, especially the PDE6 and PDE11 enzymes.^13^*In silico* studies play an important role in better understanding of the molecular determinants of drug–PDE5 binding interactions.

In the present study, a series of chromeno[2,3-*c*]pyrrol-9(2*H*)-one were studied, and these derivatives exhibit potent inhibitory potency, remarkable selectivity and excellent pharmacokinetic properties, which may serve as a potential candidate for the treatment of PAH.^14,15^ An integrated computation approach is carried out to characterize the microscopic interaction and binding mechanism between PDE5 and these compounds. Multiple docking strategies are used to take insight to the binding interactions, and the reliable docking results were obtained by considering the protein flexibility and the effect of polarization. The dynamics binding process of these compounds were explored by molecular dynamics (MD) simulations, and the Molecular Mechanics/Generalized Born Surface Area (MM/GBSA) binding free energy calculations and MM/GBSA binding energy decomposition analysis were employed to obtain the molecular recognition of PDE5 with the studied inhibitors. These computational results are useful for the insight into the molecular architecture of the catalytic site and the design of novel potent PDE5 inhibitors with new scaffolds.

## Materials and methods

### Protein preparation

The three-dimensional complex structure of human PDE5 with compound 57 (PDB entry: 4 MD6) was employed as the template in the docking calculations.^14^ Firstly, the crystal structure was treated with the *Protein Preparation Wizard* in Schrödinger,^16^ and the protonation states and partial charges of PDE5 were assigned using the OPLS force field.^17^ The crystal waters were removed because no crystallographic water is located in the binding pocket. Then, the minimization was terminated with the Impact Refinement module when the root-mean-square deviation (RMSD) reached a maximum cutoff of 0.30 Å. All hydrogen atoms are freely minimized to optimize H-bond network, while the heavy atoms are restrained so that the final structure dose not deviate too much from the initial geometry. The mass center of the co-crystal ligand in the crystal structure was employed to determine the location of the docking grid box.

### Ligand preparation

The chemical structures and IC_50_ values of 22 studied inhibitors are summarized in Table 1 taken from a team headed by Prof. Luo.^14,15^ Theses inhibitors with a large activity space possess a similar aryl chromeno-pyrrol scaffold. The molecular configurations of 21 inhibitors were sketched manually according to the structure of cmp57 (3-(4-hydroxybenzyl)-1-(thiophen-2-yl)chromeno[2,3-*c*]pyrrol-9(2*H*)-one) in the crystal structure. Because these inhibitors have steric hindrance with surrounding residues, each complex of PDE5-inhibitor was refined with 5000 steps of steepest descent and conjugate gradient minimization with the CHARMM force field in Discovery Studio.^18^ Then, the refined molecules were treated using the *Ligprep* module in Schrödinger, and the protonated states and tautomerization states for each molecule were generated.

**Table 1:** Chemical structures and biological activities of the studied PDE5 inhibitors

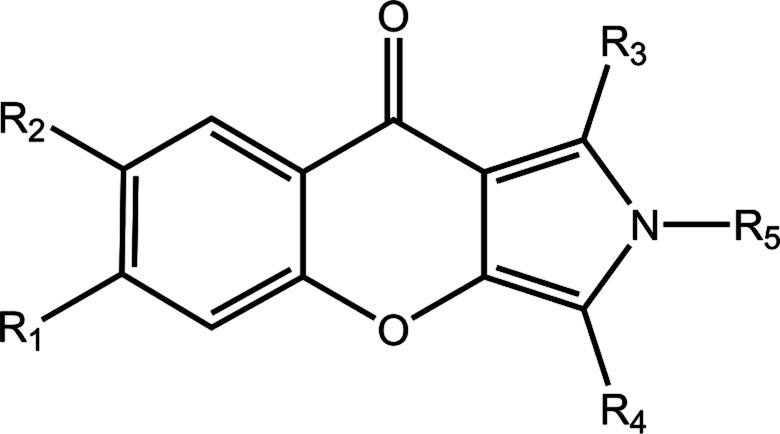							
Cpd	R_1_	R_2_	R_3_	R_4_	R_5_	IC_50_ (nM)	pIC_50_
2^a^	H	H	Thiazol-2-yl	5-Methylbenzodioxole	H	5.60	8.25
8^a^	H	H	COOCH_3_	CH_2_C_6_H_4_(*p*-OH)	H	31	7.51
17a^a^	H	H	Pyridin-2-yl	CH_2_C_6_H_4_(*p*-OH)	H	21	7.68
17b^a^	H	H	Pyrimidin-4-y	CH_2_C_6_H_4_(*p*-OH)	H	56	7.25
17c^a^	H	H	Pyridin-3-yl	CH_2_C_6_H_4_(*p*-OH)	H	77	7.11
17f^a^	H	H	5-Chloropyridin-2-yl	CH_2_C_6_H_4_(*p*-OH)	H	50	7.30
17i^a^	H	H	2-Fluoropheny	CH_2_C_6_H_4_(*p*-OH)	H	16	7.80
17j^a^	H	H	Thiazol-2-yl	CH_2_C_6_H_4_(*p*-OH)	H	5.4	8.27
19^b^	H	CH_3_	Furan-2-yl	CH_2_C_6_H_4_(*p*-O^*t*^Bu)	H	240	6.62
20^b^	H	H	Furan-2-yl	CH_2_C_6_H_4_(*p*-O^*t*^Bu)	H	1997	5.70
30^a^	H	H	Thiazol-2-yl	4-(Trifluoromethyl)benzyl	H	30	7.52
36^b^	H	H	4-Br-C_6_H_4_	CH_2_C_6_H_5_	H	319	6.50
42^b^	H	H	C_6_H_5_	CH_2_C_6_H_5_	H	77	7.11
51^b^	H	OCH_3_	Thiophen-2-yl	CH_2_C_6_H_4_(*p*-OH)	H	61	7.21
53^b^	H	CH_3_	4-F-C_6_H_4_	CH_2_C_6_H_4_(*p*-OH)	H	356	6.45
55^b^	H	Br	Thiophen-2-yl	CH_2_C_6_H_4_(*p*-OH)	H	135	6.87
57^b^	H	H	Thiophen-2-yl	CH_2_C_6_H_4_(*p*-OH)	H	17	7.77
58^b^	H	H	4-OCH_3_-C_6_H_4_	CH_2_C_6_H_4_(*p*-OH)	H	221	6.66
59^b^	H	H	Naphthalen-2-yl	CH_2_C_6_H_4_(*p*-OH)	H	456	6.34
60^b^	H	H	C_6_H_5_	CH_2_C_6_H_5_	CH_3_	902	6.04
61^b^	H	H	C_6_H_5_	CH_2_C_6_H_4_(*p*-OH)	H	18	7.74
62^b^	H	H	C_6_H_5_	CH_2_C_6_H_4_(*p*-OH)	CH_3_	137	6.86

a IC_50_ values derived from ref. 15.

b IC_50_ values derived from ref. 14.

### Molecular docking

Three docking strategies, including rigid-receptor docking (RRD), induced fit docking (IFD) and QM-polarized ligand docking (QPLD), were carried out to predict the binding mode and the rank between the binding-free energy and experimentally determined pIC_50_. In the RRD protocol, the receptor is fixed while the docked ligands are free to move, which is performed by using Glide program in the Extra Precision (XP) scoring mode. The scale factor of van der Waals radii was set as 0.8 for the protein atoms with absolute partial charges less than or equal to 0.25. The zinc and magnesium ions were assigned with a charge of 2+.

The IFD protocol was employed to consider the flexibility of both ligand and receptor. Firstly, the ligands were docked into the rigid receptor with the softened energy function in the Glide program, and the resulted top 20 poses of each ligand were retained. Then, the receptor freedom degrees are sampled with the Prime program in the Schrödinger, and the residues within 5 Å of the ligand were subjected to a conformational search and energy minimizations, although the residues outside this zone were fixed. The best receptor-ligand complex was then redocking with the default hard-potential function. The Glide XP scoring mode was employed to rank the complexes for all the docking calculations, taking into account receptor–ligand interaction energy as well as strain and solvation energies.

The quantum mechanics/molecular mechanics (QM/MM) docking was carried out with the OPLD protocol in Schrödinger so as to consider the polarization of the charge on the ligand by the receptor. Firstly, each ligand was docked by RRD protocol with standard precision (SP) scoring mode followed by XP refinement. In this step, top ten best binding poses of each ligand were generated. The polarizable ligand charges of the saved poses within the protein environment were calculated with QSite at the B3LYP/6-31G* level. At last, the ligands with QM/MM modified charges were redocked and the Emodel value was chosen to score the poses.^16^

### Molecular dynamics (MD) simulation

The docked structures of PDE5 with inhibitor 17j, 20, 57 and 59 were chosen as the starting structure for the MD simulations.^19–21^ The general AMBER force field (*gaff*)^22^ was used for the inhibitor and the ff14SB force field^23^ was used for the receptor. Each ligand was optimized by the semiempirical AM1 method, and the atomic partial charges were calculated by fitting the electrostatic potentials with the single-point Hartree–Fock (HF)/6-31G* level using the RESP technique.^24^ The Zn^2+^ and Mg^2+^ ions in the active site were calculated by the 12–6 nonbonded model *via* electrostatic and van der Waals terms implemented in MCPB tool.^25^ The whole system was immersed in a rectangular box of TIP3P water molecules,^26^ which was extended 10 Å from the solute atoms in all three dimensions. A 10 Å cutoff was chosen for the long-range electrostatic interactions and van der Waals interactions.^27,28^ The counter ions of Na^+^ were placed in the grids with the strongest negative coulombic potential region. The particle mesh Ewald (PME) method was used to treat long-range electrostatic interactions.^29^ Each complex was relaxed by 1000 cycles of steepest descent, followed by 4000 cycles of conjugated gradient minimization. The system was gradually heated from 0 to 300 K with Langevin dynamics in the NVT ensemble over a period of 100 ps. The SHAKE procedure was used to constrain all hydrogen atoms, and the time step was set to 2.00 fs.^30^ 50 ns NPT MD simulations with a target temperature of 300 K and a target pressure of 1 atm were performed. The MM optimization and MD simulations were carried out by the sander program in AMBER 16.0.

### Free energy calculation

The stable MD trajectory of each complex was extracted to evaluate the binding free energy (Δ*G*_bind_) by the MM/GBSA approach in AMBER16.^31–49^ In MM/GBSA, the binding free energy between a ligand (L) and a receptor (R) was calculated according to the following equation:^50^1
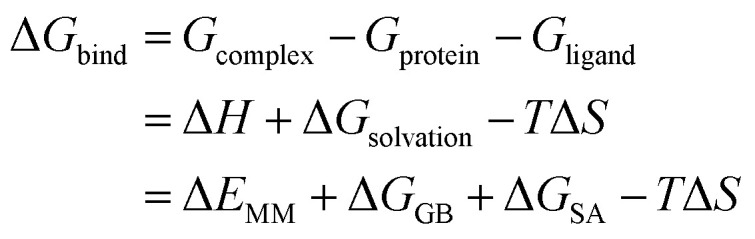


In the above equation, Δ*E*_MM_ indicates the interaction energy of protein–ligand in vacuum state, including electrostatic and van der Waals interaction energy; Δ*G*_GB_ and Δ*G*_SA_ are the polar and non-polar part of the desolvation free energy; −*T*Δ*S* is the change of conformational entropy during ligand binding, which is not considered here due to expensive computational costs and lower prediction accuracy. The electrostatic solvation energy Δ*G*_GB_ is calculated using the modified GB model.^51^ The solute and exterior dielectric constants were set to 80 and 1, respectively. The non-polar solvated free energy enthalpy was calculated wither LCPO method to consider solvent accessible surface (SASA): Δ*G*_SA_ = 0.0072 × ΔSASA.^52^ Finally, 3000 snapshots from the last 30 ns MD trajectory were chosen to evaluate all energy components.

The interaction between each residue of PDE5 and each ligand were evaluated with the MM/GBSA free energy decomposition implemented in *mm_pbsa* module.^53–57^ The residue–inhibitor interactions were divided into the following four terms: van der Waals interactions (Δ*G*_vdw_), electrostatic interactions (Δ*G*_ele_), the polar part of desolvation (Δ*G*_GB_) and the non-polar desolvation interactions (Δ*G*_SA_). The electrostatic interactions can be decomposed on per-residue basis by using the framework of GB. The non-polar part of desolvation was determined by SASA with the ICOSA program, and the other components were evaluated on the basis of the same parameters in the total free energy calculations.^53^

### Dynamic cross-correlation map (DCCM)

DCCM analysis was employed to evaluate the correlation matrix across all C_α_ atoms for the compound 17j and compound 17j systems. The correlation coefficient *S*_*ij*_ between two atoms *i* and *j* during the course of the simulation trajectory can be determined by:2
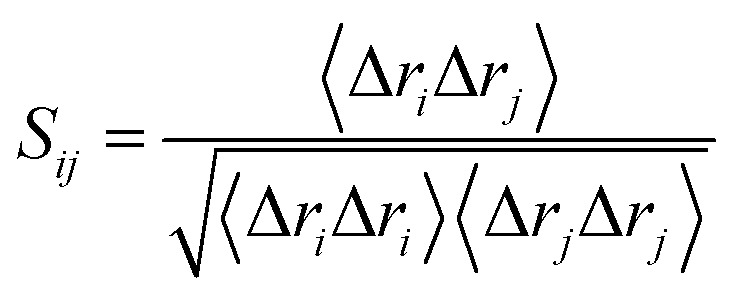
where displacement vectors Δ*r*_*i*_ or Δ*r*_*j*_ are the instantaneous fluctuation of the position of *i*th or *j*th atom with respect to its mean position, and 〈⋯〉 represents trajectory averages. Positively correlation residues move in the same direction, *i.e. S*_*ij*_ > 0, while anti-correlated residues move in the opposite direction, *i.e. S*_*ij*_ < 0.

## Results and discussion

### Comparison of three molecular docking protocols

**Fig. 1:**
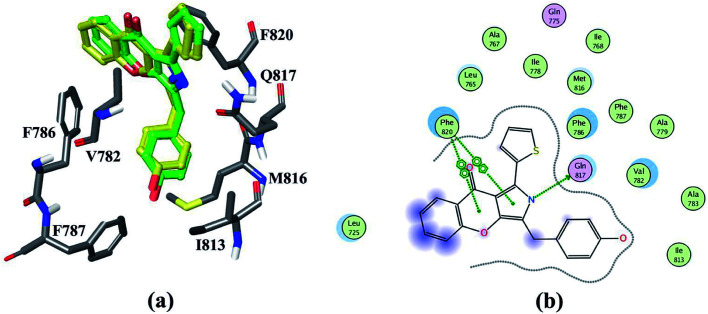
(a) The superposition of the Glide docked compound 57 and its original structure in the crystallographic complex. Carbon atoms in the crystal structure and the Glide docked conformation are colored in green and yellow, respectively; (b) schematic representation of the interactions between compound 57 and PDE5.

In order to obtain the reliably binding structure, three different docking protocols, including RRD, IFD and QPLD were compared. The docking performance of RRD was firstly evaluated by redocking the co-crystallized compound 57 to the active site of PDE5. As shown in Fig. 1a, the crystal structure of compound 57 agrees well with its docked pose with the lowest docking score by RRD, and the root mean square deviation (RMSD) of them is 0.76 Å for only the heavy atoms, and is 1.08 Å for all atoms. Fig. 1 presents the interaction of the docked binding mode of compound 57, and the key features for ligand binding are well reproduced. The secondary amine of compound 57 forms a key hydrogen bond interaction with the carbonyl oxygen of main chain of Gln817. The aryl-chromeno-pyrrol ring of compound 57 forms aryl–aryl interactions with the phenyl ring of Phe786 and Phe820 in a T-shaped geometry. Compound 57 can form hydrophobic interaction with Leu765, Ile768, Val782, Phe787, Ile813 and Met816.

In the physical conditions, both ligand and receptor undergo movements to adjust their conformations for accommodating each other, which is termed as “induced fit”. Compared to RRD, IFD can take into account the flexibility of PDE5 within 5 Å residues of ligand during their binding process, and thus IFD obtain better performance to determine the binding mode of the docked compound 57. The RMSD value between the docked pose predicted by IFD and the cocrystallized one is only 0.57 Å for heavy atoms and 0.93 Å for all atoms.

**Fig. 2:**
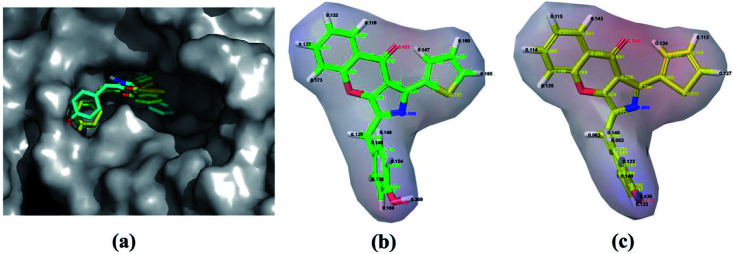
(a) Solvent accessible surface of the binding pocket of PDE5 with the docking conformation of compound 57 by RRD, IFD and QPLD. Carbon atoms are colored in green, cyan and yellow, respectively. (b) and (c) Electrostatic potential value plotted on the Connolly surface of compound using electrostatic potential fitting charge (ESP) atomic charges derived from a B3LYP/6-31G* and OPLS force field.

QPLD was also employed to improve the accuracy of partial charges on the ligand atoms, which was derived from quantum mechanical calculations, and the ligand was redocked into the binding site of receptor. Compared to RRD, the redocked compound 57 predicted by QPLD superimpose well with the crystal structure, and the RMSD of them is 0.48 Å for heavy atoms and 0.79 Å for all atoms. Fig. 2a illustrates the molecular surface of PDE5 with binding pose of compound 57 predicted by three docking protocols, and Fig. 2 shows the difference of the electrostatic potential surface with the polarizable ligand charge derived by ESP and OPLS force field.

**Fig. 3:**
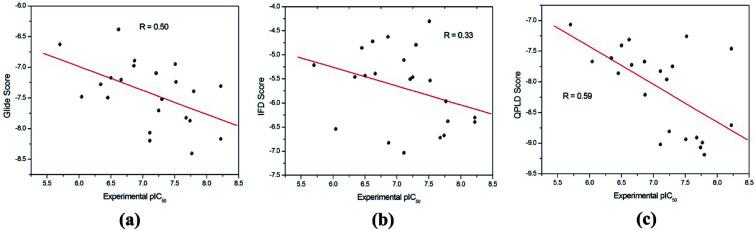
The correlation between the experimental pIC_50_ and the docking scores predicted by (a) RRD, (b) IFD and (c) QPLD.

The binding modes of the whole dataset were explored by three docking protocol, and their docking scores are summarized in Table 2. The relative binding affinities of theses inhibitors can be evaluated by constructed correlation modes between the docking score and the experimental pIC_50_. According to Fig. 3, the correlation coefficients (*r*) for RRD, IFD and QPLD are 0.50, 0.33 and 0.59 respectively. Compared with IFD, RRD and QPLD have better docking performance. Although the protein flexibility within the binding site was considered, IFD do not exhibit better capability to rank the bioactivities of these ligands than RRD, which shows that IFD may be not the best choice for studied inhibitors of PDE5. QPLD achieves the best performance in fast ranking of these inhibitors, indicating that the accuracy of electric charges plays an important role in protein–ligand docking process.

**Table 2:** Docking score predicted by three different docking protocols

Cpd	pIC_50_	Glide score	IFD score	QPLD score
2	8.22	−7.31	−6.39	−7.46
8	7.51	−6.95	−4.30	−8.94
17a	7.68	−7.82	−6.72	−8.91
17b	7.25	−7.70	−5.46	−8.81
17c	7.11	−8.20	−7.03	−9.02
17f	7.30	−7.52	−4.79	−7.75
17i	7.80	−7.39	−6.38	−9.19
17j	8.22	−8.17	−6.30	−8.71
19	6.62	−6.38	−4.72	−7.31
20	5.70	−6.63	−5.21	−7.07
20	5.70	−6.63	−5.21	−7.07
36	6.50	−7.17	−5.43	−7.41
42	7.11	−8.07	−5.11	−7.83
51	7.21	−7.10	−5.50	−7.96
53	6.45	−7.50	−4.86	−7.86
55	6.87	−6.89	−6.82	−8.21
57	7.77	−8.41	−5.96	−8.99
58	6.66	−7.20	−5.39	−7.72
59	6.34	−7.28	−5.46	−7.61
60	6.04	−7.48	−6.54	−7.67
61	7.74	−7.87	−6.67	−9.07
62	6.86	−6.98	−4.63	−7.67

### Molecular dynamics simulations and MM/GBSA calculations

**Fig. 4:**
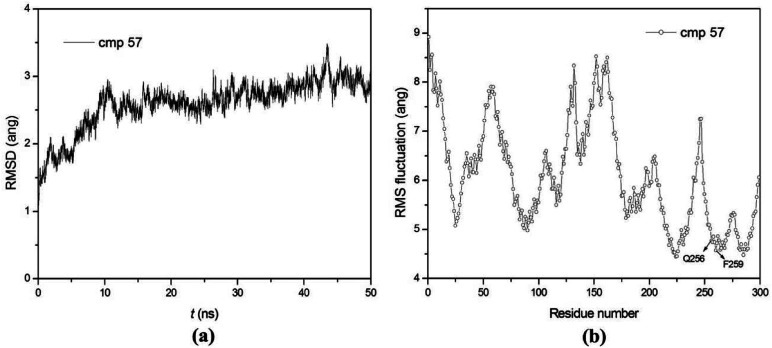
(a) RMSD of the backbone C_α_ atoms of the PDE5-compound 57 complexes with respect to the first snapshots as a function of time. (b) RMSF of backbone atoms *versus* residue number of PDE5–compound 57 complex. For simplicity, all residues are sequentially renumbered from 1 to 299.

50 ns MD simulations were carried out for PDE5 with four representative inhibitors (17j, 20, 57 and 59) so as to explore the dynamic interaction patterns. The structural determinant of the substitutions at the R_3_ and R_4_ position were characterized with the compare of the two inhibitors in each pair (17j *vs.* 59, 20 *vs.* 57). The RMSD of the compound 57 relative to the starting structure C_α_ atom in the production phase is shown in the Fig. 4a, indicating that the system reaches equilibrium near 30 ns and the average C_α_ RMSD of the compound 57 is 2.59 ± 0.37 Å. Then, the root mean square fluctuation (RMSF) *versus* the residue number for compound 57 was analyzed. As illustrated in Fig. 4b, there are relatively small fluctuations with the region around residues Gln256 and Phe259 as a result of direct interaction with the inhibitor.

Furthermore, the binding free energies of the four systems were evaluated by MM/GBSA free energy calculation, which exhibits good performance in the ranking of experimental binding affinities. According to Table 3, the compound 17j system has the strongest binding affinity (Δ*G*_pred_ = −34.35 kcal mol^−1^), and the binding affinity of compound 57 system is slightly weaker than that of the system (Δ*G*_pred_ = −33.65 kcal mol^−1^). All of them have stronger binding affinity in comparison with compound 59 and 20 systems (Δ*G*_pred_ = −29.70 kcal mol^−1^ and Δ*G*_pred_ = −30.61 kcal mol^−1^), respectively, which are in good agreement with the experimental data. According to the energy components of the binding free energies (Table 3), the non-boned contribution composed of the electrostatic and van der Waals terms, are the mainly favorable contribution to ligand binding, while the polar solvation term oppose binding. The non-polar solvation term corresponding to the burial of SASA upon ligand binding is slightly contribution.

**Table 3:** The predicted binding free energies and the individual energy components for the studied systems (kcal mol^−1^)

System	Polar contributions		Nonpolar contributions		Δ*G*_pred_
	Δ*E*_ele_	Δ*G*_GB_	Δ*E*_vdw_	Δ*G*_SA_	
cmp 17j	−18.46 ± 0.38	30.83 ± 0.40	−41.57 ± 0.51	−5.15 ± 0.13	−34.35 ± 0.57
cmp 20	−9.31 ± 0.78	28.39 ± 0.37	−43.41 ± 0.32	−6.28 ± 0.05	−30.61 ± 0.41
cmp 57	−13.35 ± 0.49	32.68 ± 0.32	−46.57 ± 0.51	−6.40 ± 0.17	−33.65 ± 0.58
cmp 59	−12.84 ± 0.32	29.24 ± 0.29	−40.64 ± 0.44	−5.46 ± 0.33	−29.70 ± 0.53

### The important residues for PDE5–studied inhibitor interactions

**Fig. 5:**
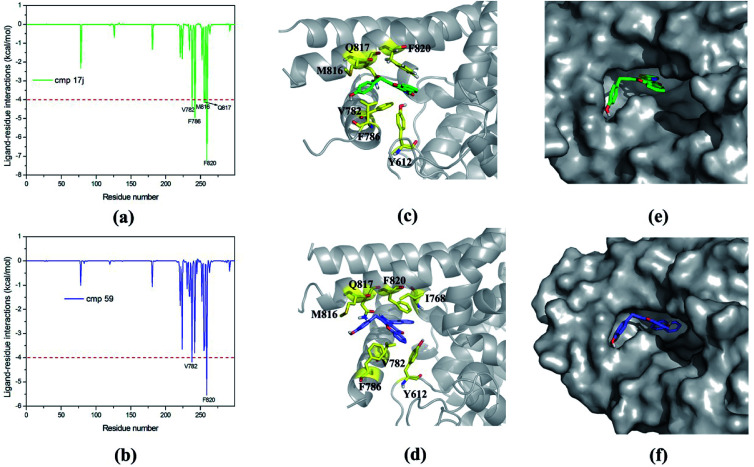
Inhibitor–residue interaction spectrums for (a) PDE5/cmp17j complex, (b) PDE5/cmp59 complex. Comparison of the averaged structures for (c) PDE5/cmp17j and (d) PDE5/cmp59. Carbon atoms of the ligands are colored in green and purple. Solvent accessible surface of the binding pocket of PDE5 for (e) cmp17j and (f) cmp59.

Based on MM/GBSA, the free energy decomposition was used to obtain the residue–inhibitor interaction spectra so as to have a deeper insight into protein–inhibitor interaction patterns. Some important information may be obtained for future rational design of more potent PDE5 inhibitors by comparing the representative inhibitors with different types of substituents. Fig. 5 illustrates the computational results and binding modes for compound 17j and compound 59. Table S1† represents the numerical data of the contributions of important residues to the ligand binding. As shown in Table 1, the only difference of compound 17j and 59 exits at R_3_, where the former possesses a thiazole ring and the latter possesses naphthalene ring leading to the 84-fold difference in activity. According to Table 2, the predicted binding free energy for compound 17j is stronger than that for compound 59 by ∼5 kcal mol^−1^, and the electrostatic interaction term determine the difference of the binding affinities of them. Based on the interaction spectrums (Fig. 5), the residues of Val782, Phe786, Met816, Qln817 and Phe820 have the largest contribution to compound 17j binding to PDE5. The secondary amine of compound 17j can form a key hydrogen bond with the amide oxygen of the Gln817, and the distance between the nitrogen atom of the pyrrol ring of compound 17j and the amide oxygen is 1.75 Å in comparison with 2.52 Å for compound 59, as a result that the Qln817 contributes −4.14 kcal mol^−1^ for compound 17j, mainly coming from electrostatic interaction energy term (−5.64 kcal mol^−1^) on the basis of energy decomposition analysis (Table S1†). The residue Qln817 contributes −3.50 kcal mol^−1^ for compound 59, and the electrostatic energy term is −3.94 kcal mol^−1^. In addition, the thiazole ring of compound 17j can form polar interaction with the phenolic hydroxyl group of Tyr612. The residue Tyr6112 contributes −2.32 kcal mol^−1^ for compound 17j, mainly coming from the electrostatic interaction (−1.32 kcal mol^−1^) whereas the Tyr612 contributes −1.00 kcal mol^−1^ for compound 59 primarily through the electrostatic interaction energy term (−0.24 kcal mol^−1^). According to Fig. 5c, compound 17j can form aryl–aryl interactions in a T-shaped geometry with the phenyl ring of Phe786 and Phe820, while compound 59 can form a parallel, stacked arrangement with the phenyl ring of Phe820. These computational results are in accordance with the previous study that hydrogen bond with Gln817 and the π–π stack against Phe820 are two conserved characteristic for many PDE inhibitors.^8^ The residue Phe786 and Phe820 contributes −4.96 kcal mol^−1^ and −7.20 kcal mol^−1^ for compound 17j, mainly coming from the van der Waals interaction energy term (−4.92 kcal mol^−1^ and −7.74 kcal mol^−1^), and the residue Phe786 and Phe820 contributes −3.80 kcal mol^−1^ and −5.54 kcal mol^−1^ for compound 59. The aryl-chromeno-pyrrol ring of compound 17j can also form hydrophobic interaction with the nonpolar residue Val782 and Met816, and the energy contributions of them are −4.38 kcal mol^−1^ and −4.12 kcal mol^−1^, mainly coming from the van der Waals interaction energy term (−4.06 kcal mol^−1^ and −3.80 kcal mol^−1^).

**Fig. 6:**
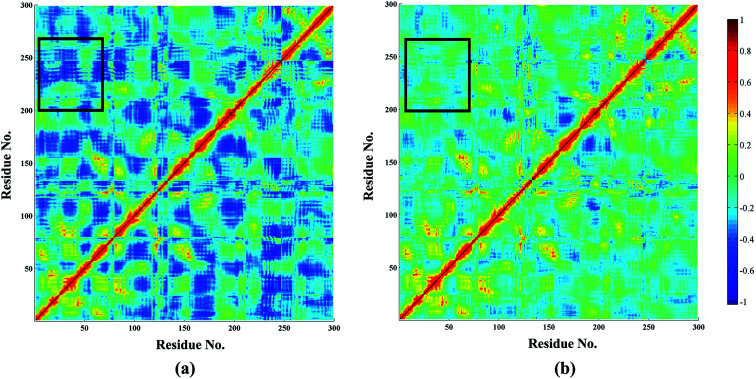
Dynamic cross-correlation map (DCCM) analyses of PDE5, for the (a) cmp17j and (b) cmp59. The color scale is shown on the right changing from red (highly positive correlations) to blue (highly negative correlations).

In addition, dynamic cross-correlation maps were employed to evaluate the conformational changes of PDE5/cmp17j and PDE5/cmp59 (Fig. 6).^58^ As shown in Table 3, the compound 17j system has the strongest binding affinity (Δ*G*_pred_ = −34.35 kcal mol^−1^) than compound 59 system (Δ*G*_pred_ = −29.70 kcal mol^−1^), which suggests that the compound 59 system may exhibit more fluctuations. Small values are found around residues 20–50 and residues 230–260 in the case of the compound 17j system, while these coupling obviously increase in the compound 59 system, which indicates that the flexibility of the compound 59 system is coupled with other motions. All of them are conformational couplings in the different helices and β-strands, including α1 with α2 and β1. In general, the compound 59 system has relatively higher correlation than the compound 17j system resulting from the poor receptor–ligand binding affinity of the former in comparison with the latter.

**Fig. 7:**
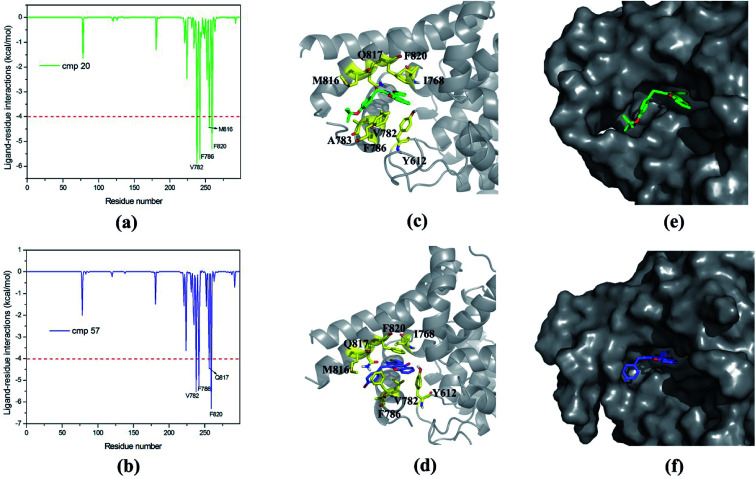
Inhibitor–residue interaction spectrums for (a) PDE5/cmp20 complex, (b) PDE5/cmp57 complex. Comparison of the averaged structures for (c) PDE5/cmp20 and (d) PDE5/cmp57. Carbon atoms of the ligands are colored in green and purple. Solvent accessible surface of the binding pocket of PDE5 for (e) cmp20 and (f) cmp57.

The mainly difference of compound 20 and 57 exits at R_4_ (Table 1), where the latter possesses a phenolic hydroxyl and the hydrogen atom was substituted with tertiary butyl for the former resulting in a 117-fold difference in activities. As shown in Table 2, the predicted binding free energy for compound 20 is weaker than that for compound 57 by ∼3 kcal mol^−1^, and the electrostatic interaction term determines the difference of the binding affinities of them. In Fig. 7, the amide of Gln817 can form two hydrogen bonds with the secondary amine and thiophen ring of compound 57, while the amine oxygen of the Gln817 forms a hydrogen bond with the secondary amine of compound 20. The energy decomposition analysis (Table S1†) illustrates that the Qln817 contributes −4.46 kcal mol^−1^ for compound 57, mainly coming from electrostatic interaction energy term (−4.30 kcal mol^−1^). The residue Qln817 contributes −2.18 kcal mol^−1^ for compound 20, and the electrostatic energy term is −2.36 kcal mol^−1^. In addition, the hydroxyl group of compound 57 can form polar interaction with Qln817 and Ile813. The energy contribution of Ile813 for compound 57 is −2.50 kcal mol^−1^ in comparison with −1.58 kcal mol^−1^ for compound 20, mainly coming from the electrostatic energy term (−2.40 kcal mol^−1^). The aryl-chromeno-pyrrol ring of compound 57 can form π–π stack against phenyl ring of Phe786 and Phe820. The residue Phe786 and Phe820 contributes −5.26 kcal mol^−1^ and −6.30 kcal mol^−1^ for compound 57, mainly coming from the van der Waals interaction energy term (−5.66 kcal mol^−1^ and −7.18 kcal mol^−1^). The compound 57 can also form hydrophobic interaction with the nonpolar residue Ile768, Val782, Phe786 and Met812, the energy contributions of Val782 is −5.52 kcal mol^−1^, primarily through the van der Waals interaction energy term (−5.12 kcal mol^−1^).

## Conclusion

In this work, we intended to characterize the binding modes between PDE5 with a serious of aryl chromeno-pyrrol analogs by using molecular docking protocols, MD simulations and free energy calculations. The comparison of the performance of three different docking protocols indicates that QPLD achieves better accuracy of prediction by considering polarization of the charge on the ligand in the field of receptor in comparison with RRD and IFD. The dynamic bind process and structural determinants were explored by MD simulations, MM/GBSA free energy calculations and free energy decomposition for the compounds 17j, 20, 57 and 59, which are in accordance with the experimental data. The structural and energetic results obtained here shed light on some important guidance for the rational design of novel potent PDE5 inhibitors.

## Conflicts of interest

The authors declare no competing financial interests.

## Abbreviation

PDE5: Phosphodiesterase type 5
cGMP: Cyclic guanosine monophosphate
RRD: Rigid-receptor docking
IFD: Induced fit docking
QPLD: QM-polarized ligand docking
QM/MM: Quantum mechanics/molecular mechanics
MD: Molecular dynamics
MM/GBSA: Molecular mechanics/generalized Born surface area
GAFF: General AMBER force field
PME: Particle mesh ewald
SASA: Solvent accessible surface area
DCCM: Dynamic cross-correlation maps
RMSD: Root-mean-square deviation
RMSF: Root mean square fluctuation

## Supplementary Material

RA-008-C8RA06405A-s001
