# Supplementary material for: Exploring the binding mechanisms of PDE5 with chromeno[2,3-c]pyrrol-9(2H)-one by theoretical approaches
Source: RSC Adv. 2018 Aug 29;8(53):30481–90. doi: 10.1039/c8ra06405a (PMC9085377; doi:10.1039/c8ra06405a)
Supplement: RA-008-C8RA06405A-s001 [file RA-008-C8RA06405A-s001.pdf]

Electronic Supplementary Material (ESI) for RSC Advances.
This journal is © The Royal Society of Chemistry 2018

# Supporting Information

**Table S1**. Binding free energy contributions of key binding-site residues calculated by the binding energy decomposition for three representative compounds( kcal/mol).

| | cmp 17j | | | | | cmp 20 | | | | |
|---|---|---|---|---|---|---|---|---|---|---|
| | $\Delta E_{vdw}$ | $\Delta E_{ele}$ | $\Delta G_{GB}$ | $\Delta G_{SA}$ | $\Delta G_{bind}$ | $\Delta E_{vdw}$ | $\Delta E_{ele}$ | $\Delta G_{GB}$ | $\Delta G_{SA}$ | $\Delta G_{bind}$ |
| Tyr612 | -2.10 | -1.32 | 1.50 | -0.40 | -2.32 | -1.50 | -1.16 | 1.32 | -0.30 | -1.64 |
| Ile768 | -1.68 | -0.10 | 0.08 | -0.14 | -1.82 | -2.26 | -0.14 | 0.08 | -0.14 | -2.46 |
| Val782 | -4.06 | -0.02 | 0.02 | -0.32 | -4.38 | -5.18 | -0.01 | 0.20 | -0.50 | -5.58 |
| Phe786 | -4.92 | -0.50 | 1.46 | -1.00 | -4.96 | -5.50 | -0.86 | 1.64 | -0.98 | -5.70 |
| Phe787 | -2.12 | -0.08 | 0.16 | -0.02 | -2.38 | -0.92 | -0.20 | 0.58 | -0.06 | -0.60 |
| Ile813 | -1.86 | -0.50 | 0.68 | -0.18 | -1.86 | -1.46 | -0.18 | 0.28 | -0.20 | -1.58 |
| Met816 | -3.80 | -0.48 | 0.70 | -0.54 | -4.12 | -4.22 | -0.10 | 0.48 | -0.62 | -4.44 |
| Gln817 | -3.02 | -5.64 | 4.78 | -0.24 | -4.14 | -2.32 | -2.36 | 2.76 | -0.24 | -2.18 |
| Phe820 | -7.74 | -0.74 | 0.58 | -0.78 | -7.20 | -5.78 | -0.48 | 0.82 | -0.78 | -5.30 |

| | cmp 57 | | | | | cmp 59 | | | | |
|---|---|---|---|---|---|---|---|---|---|---|
| | $\Delta E_{vdw}$ | $\Delta E_{ele}$ | $\Delta G_{GB}$ | $\Delta G_{SA}$ | $\Delta G_{bind}$ | $\Delta E_{vdw}$ | $\Delta E_{ele}$ | $\Delta G_{GB}$ | $\Delta G_{SA}$ | $\Delta G_{bind}$ |
| Tyr612 | -1.98 | -1.20 | 1.44 | -0.26 | -2.00 | -1.44 | -0.24 | 0.88 | -0.22 | -1.00 |
| Ile768 | -3.18 | -0.46 | 0.30 | -0.30 | -3.62 | -3.32 | -0.08 | 0.02 | -0.32 | -3.64 |
| Val782 | -5.12 | -0.20 | 0.06 | -0.54 | -5.52 | -3.58 | -0.20 | 0.12 | -0.50 | -4.18 |
| Phe786 | -5.66 | -0.18 | 1.14 | -0.54 | -5.26 | -3.64 | -0.38 | 1.10 | -0.88 | -3.80 |
| Phe787 | -1.40 | -0.02 | 0.46 | -0.14 | -1.06 | -1.34 | -0.02 | 0.14 | -0.08 | -1.32 |
| Ile813 | -2.40 | -0.20 | 0.24 | -0.18 | -2.50 | -1.50 | -0.32 | 0.40 | -0.24 | -1.66 |
| Met816 | -2.08 | -0.12 | 0.40 | -0.12 | -1.94 | -3.56 | -0.32 | 0.74 | -0.56 | -3.70 |
| Gln817 | -3.98 | -4.30 | 4.30 | -0.48 | -4.46 | -3.38 | -3.94 | 4.32 | -0.52 | -3.50 |
| Phe820 | -7.18 | -0.58 | 0.90 | -0.58 | -6.30 | -5.82 | -0.42 | 0.76 | -0.90 | -5.54 |
